# Supplementary figures and images for: Lamina-specific AMPA receptor dynamics following visual deprivation in vivo
Source: eLife. 2020 Mar 3;9:e52420. doi: 10.7554/eLife.52420 (PMC7053996; doi:10.7554/eLife.52420)

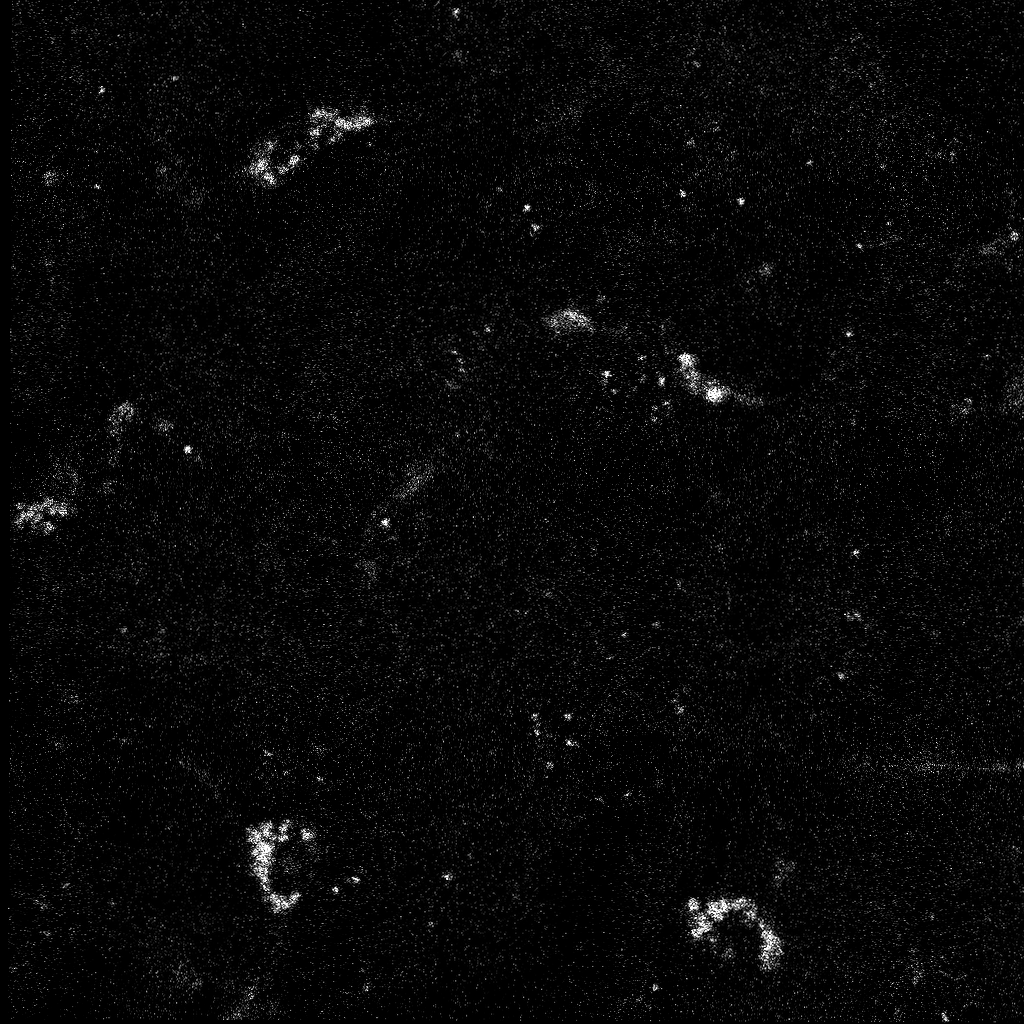

Supplement: Supplementary file 1. — Channel 1 (SEP-GluA1 signal). [file elife-52420-supp1.zip › Supplemental image 2.tif]

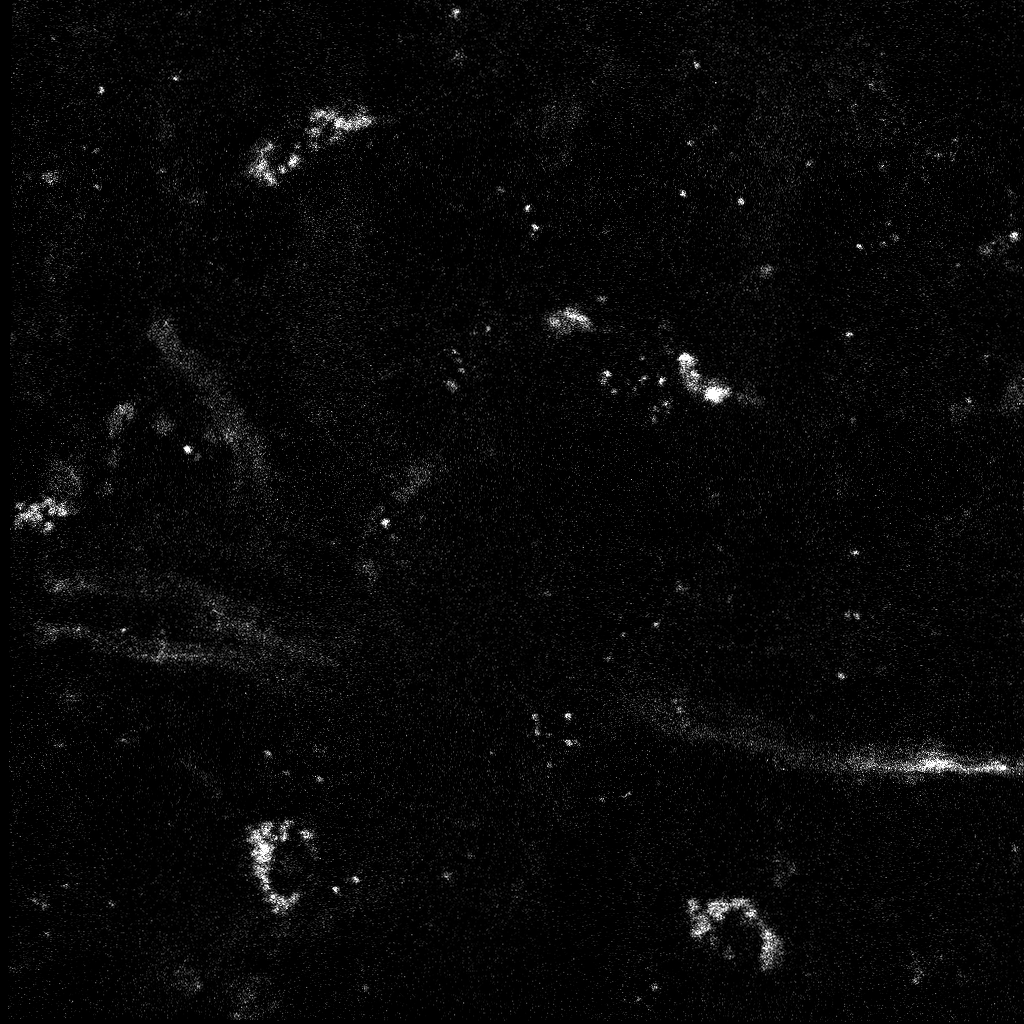

Supplement: Supplementary file 2. — Channel 2 (dsRed2 signal). [file elife-52420-supp2.zip › Supplemental image 1.tif]
